# Valorization of Agri-Food Wastes as Sustainable Eco-Materials for Wastewater Treatment: Current State and New Perspectives

**DOI:** 10.3390/ma14164581

**Published:** 2021-08-15

**Authors:** Ecaterina Matei, Maria Râpă, Andra Mihaela Predescu, Anca Andreea Țurcanu, Ruxandra Vidu, Cristian Predescu, Constantin Bobirica, Liliana Bobirica, Cristina Orbeci

**Affiliations:** 1 Faculty of Materials Sciences and Engineering, University POLITEHNICA of Bucharest, 060042 Bucharest, Romania; ecaterina.matei@upb.ro (E.M.); maria.rapa@upb.ro (M.R.); rvidu@ucdavis.edu (R.V.); 2 Center for Research and Eco-Metallurgical Expertise, Faculty of Materials Science and Engineering, University POLITEHNICA of Bucharest, 060042 Bucharest, Romania; anca.turcanu@upb.ro; 3 Department of Electrical and Computer Engineering, University of California Davis, One Shields Avenue, Davis, CA 95616, USA; 4 Faculty of Applied Chemistry and Materials Science, University POLITEHNICA of Bucharest, 060042 Bucharest, Romania; constantin.bobirica@upb.ro (C.B.); liliana.bobirica@upb.ro (L.B.); cristina.orbeci@upb.ro (C.O.)

**Keywords:** agri-food wastes, eco-materials, wastewater treatment

## Abstract

The paper addresses environmental protection by valorizing an important agri-food waste category, namely fruit and vegetables with focusing on the main characteristics regarding consumption, waste quantities, and ways for valorizing these materials. Thus, vast research was undertaken in order to emphasize the main commodities and their potential application as adsorbents for organic and inorganic pollutants. The main methods or treatment techniques applied for the valorization of eco-materials as adsorbents were presented and the principal efficiency results were indicated. The advantages and disadvantages of using these eco-materials as adsorbents in wastewater treatment were revealed and future recommendations were established. According to the international statistics, the most purchased and consumed five commodities were studied regarding waste generations as potential conversion into eco-materials with an adsorbent role for water pollutants. Thus, the performances for adsorbents based on fruit wastes (such as citrus, banana, apples, grapes, mango) and vegetable wastes (such as potatoes, tomatoes, cabbage, carrots, cauliflower, and/or broccoli) were studied and highlighted in this research.

## 1. Introduction 

Nowadays, the ecosystem’s health and life quality are strongly determined by the wastes quantities that are gradually increasing. At a global level, the agro-industry generates valuable materials as agri-food wastes with well-known potential. These possess natural bioactive compounds with utilization in the food, pharmaceutical, and cosmetics industry or composting, energy, and bioethanol production [1,2,3,4,5]. However, a large quantity of agri-food wastes linked with economic pressure on resource sustainability requires more attention concerning the agro-industrial waste use and recycling methodologies [6]. In the last 50 years, agricultural activities have become more intense and the use of fertilizers and pesticides have given rise to inequality between productivity and environmental factors, and soil requiring more organic matter. In these circumstances, agricultural wastes could be integrated by clean technologies or be returned as an organic substrate to the soil as valuable materials, with minimum risk to environmental factors and ecosystems. Moreover, these materials could be used as animal food, combustion raw materials, or disposed to landfills, but with high implications on environmental issues. Due to their microbial decomposition agri-food wastes could be associated with some potential risks for the environment and their treatment is compulsory [7].

As a rule, agri-food wastes have a complex structure and composition consisting of polysaccharides, proteins, carbohydrates, polyphenolic constituents, etc. [8,9]. These properties could offer the possibility of using them as renewable natural resources. In addition, agri-food wastes are inexpensive, easy to access, ecofriendly, and sustainable. Under these considerations, these types of wastes are evaluated as eco-materials. Applying different physical and chemical treatments and activation methods, the agri-food wastes could be converted into a carbonized biomass (charcoal) with well-developed microporosity and functional groups at the surface acting as sorbents [10,11,12] or ion exchangers [13,14,15,16,17,18,19]. The adsorption capacity of these low-cost adsorbents is comparable to the commercially activated carbon having roles in organic and inorganic pollutants removal from synthetic solutions and wastewaters [20]. Olivera et al. identified five categories of sorbents obtained from agri-food wastes: activated carbons, biosorbents, bone char, adsorbents with chitin, and chitosan content, and ion exchange resins [7]. Given their origins, agri-food wastes, and especially fruit and vegetable wastes, were tested for different applications such as bioactive compounds used for obtaining, discoloring of sugar and vegetable oils, and removal of copper ions from the beverage industry [7].

**Figure 1:**
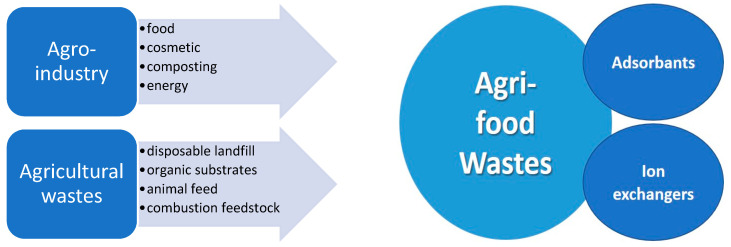
The main sources of agri-food wastes and their applications.

Agro-industrial wastes represent a feasible solution for environmental conservation and natural resources protection. Their composition offers a large spectrum of applications. Figure 1 illustrates the main reuse directions of agri-food wastes as valuable materials. 

The agri-food industrial sector generates important quantities of agricultural wastes. In their studies, Garske et al. stated that food could be wasted at the household level, with a negative impact on natural resources and the environment [21]. A way to take advantage of these wastes as inexpensive and sustainable materials is to convert them into low-cost adsorbents. So far, there were many research papers published in recent years, with a focus on inorganic and organic pollutants removal, and some review papers [22,23,24,25] reveal significant information and lead to a comprehensive understanding of the potential use of these agri-food wastes as low-cost adsorbents. 

A recently published article estimated that 180 kg of food per person and per year (kg/p/y) are wasted annually in the EU, keeping in mind that the food wastes generated from the preparation to consumption stage and that the households are the highest contributor to food waste generation. The study points out that EU households have the highest participation in food waste production. The quantity of wastes resulted annually from fruits and vegetables is approximated at 35.3 kg per person per year, from which 14.2 kg could be avoidable [26]. According to these studies, the quantified food waste was 123 kg/p/y, in the case of households and food services [27]. With respect to the fruit and vegetables (including potatoes) commodities, almost 63% results as food wastes. These commodities, representing about one-third of food and exposed inedible parts (such as peel, seeds, etc.) are short-lived and low-cost. The literature indicates differences between the quantity of avoidable and unavoidable wastes from country to country, based on consumers’ behavior, possibly due to the cultural and economic components. 

The utilization of different agricultural wastes as eco-materials with adsorption capacities for the water and wastewater treatment, dedicated to organic and inorganic pollutants removal, linked with the efficiencies, and regeneration performances data for analyzed fruit and vegetables wastes are summarized in this review paper. In addition, different functionalized or treated method wastes and mechanisms of pollutants removal are presented, where it was applicable as the current state of research. Based on this synthesis, the highest development, and future directions and needs, are discussed on the preparation of novel adsorbents as eco-materials using fruit and vegetable wastes. All information is focused on the top five commodities regarding both fruits (citrus, banana, apples, grapes, and mango) and vegetables (potatoes, tomatoes, cabbage, carrots, cauliflower, and/or broccoli) consumption according to the Food and Agriculture Organization of the United Nations (FAO) statistics, as mainly waste generation sources. Conclusions are based on the literature reviewed. The paper also establishes the future research perspectives in the field of adsorbents utilization when agricultural wastes are used for water treatment applications.

## 2. Agri-Food Waste Quantification

There are different definitions regarding agri-food wastes. Considering the definition of FAO, food waste is described as food that is not suitable for human consumption after expiring or becoming tainted. This definition is supported by the food degradation but could also be due to the oversupply on the market or consumer eating habits. The other definition given by The Food Use for Social Innovation by Optimizing Waste Prevention Strategies (FUSIONS) project, funded by the European Commission, refers to the food waste as part of ”food and spoiled parts of food removed from the food supply chain” to be recovered or stored (composting, anaerobic digestion, co-generation, incineration, etc.) [28].

Furthermore, Kummu and colleagues found differences between food losses and food wastes, the first one referring to production, postharvest, and processing of products, and the second one being associated with distribution and consumption activities [29].

As the origin, agri-food wastes are generated from (a) agricultural, (b) forest, (c) municipal, and (d) industrial activities. All of these wastes are of the organic type and can include livestock slurry, manure, pruning, and maintenance activities of woodlands to degummed fruits and legumes, bagasse, sludge from wool, cellulose, etc.

As crops, sugarcane (21% in 2018), maize (13% in 2018), rice (9% in 2018), and wheat (8% in 2018) are the most produced worldwide by quantity [30]. It has been estimated that rice, corn, and wheat represent together over 33% of all calories spent by the world population, while fruit and vegetables represent over 23%. As wastes, these quantities are expected to grow and are susceptible to have harmful effects on the environment [31]. Raw materials obtained from rice straw, rice husk, corncob, and wheat straw might be converted into valuable products such as bioethanol [32,33], biomass-degrading enzymes [34], biogas [35], antioxidants [36], adsorbents [37], lactic acid [38], husk charcoal briquettes or husk charcoal for replacement of fossil fuel, and anticoagulants [39]. Moreover, some of these materials are also used for eco-friendly composites production, such as waste-wood [40].

Lignin and cellulose have as primary components –OH and –COOH functional groups that can substitute the H^+^ with As(III), Cd(II), Cr(IV), Hg(II), Pb(II), and Ni(II) as metalloids and metal ions in order to generate complexes, as it was tested for grape and apple wastes, grounds from tea and coffee, nutshells, leaves, algae, rice, and sunflower plants [41]. In order to enhance the adsorption capacity and reinforce the functional groups potential of these agricultural waste-derived adsorbents, their structure can be modified by different physical and chemical pre-treatments, obtaining carbonaceous materials (biochar), with high surface area and pore volume [42,43].

The forest wastes, such as hardwoods (oak, maple, hickory, and birch) and softwoods (pine, spruce, fir, and juniper) represent two major types of wood waste that could be valorized as important products for obtaining active packaging for protection against oxidative damage of food [44]. In addition, different active components from wood wastes could be reintegrated in oil-based products, such as ethanol, lignin products, and vanillin [31]. Usually, wood wastes (at small sizes as chips or fibers) could be converted into new materials such as composites using “cascade” applications as efficient resources conservation and carbon storage actions [40].

The processing of food and vegetables is responsible for the generation of agri-food wastes. According to Tokusoglu [45], the production and conservation of fruits and vegetables produce almost 14.8% of food waste derivatives, being the third industry, after the beverage industry (26%) and cheese, milk, and ice cream industry (21.3%). With a lower percentage, the fabrication of starch and grain crops (12.9%), oils and fats (3.9%), the production and processing of meat products (8%), and fish and fish commodities (0.4%) are also contributors as agri-food waste generation.

Ben-Othman and colleagues stated that agricultural wastes (almost reaching up to 50%) are responsible for negative environmental impacts [46]. The FAO report identified four hotspots in terms of food wastages (the term “wastage” comprises both food loss and waste) related to significant environmental issues. These hotspots refer mainly to the carbon footprint evaluated from wastage of cereals (34% of total), especially from the high quantities of rice wastage, wastage of meat (21% of total) with substantial impact on the environment concerning land occupation, fruit wastage (6% of total) caused by food wastage volumes, and vegetable wastage (21% of total), mainly due to large wastage volumes [47]. According to other publications, the FAO estimated that the most amount of food that is wasted or lost refers to the fruit, vegetables, and seafood industry, and represents one-third of all the produced food [46,48]. Among agri-food wastes, fruit and vegetable commodities induce critical losses and wastes quantities, both in fresh and processing industries, turning into a serious threat regarding nutritional, economical, and environmental issues. It was evaluated that the losses and wastes generated by fruits and vegetables total 60% from all types of foods and processing operations covering about 25% to 30% of wastes from the whole commodity group [49].

**Figure 2:**
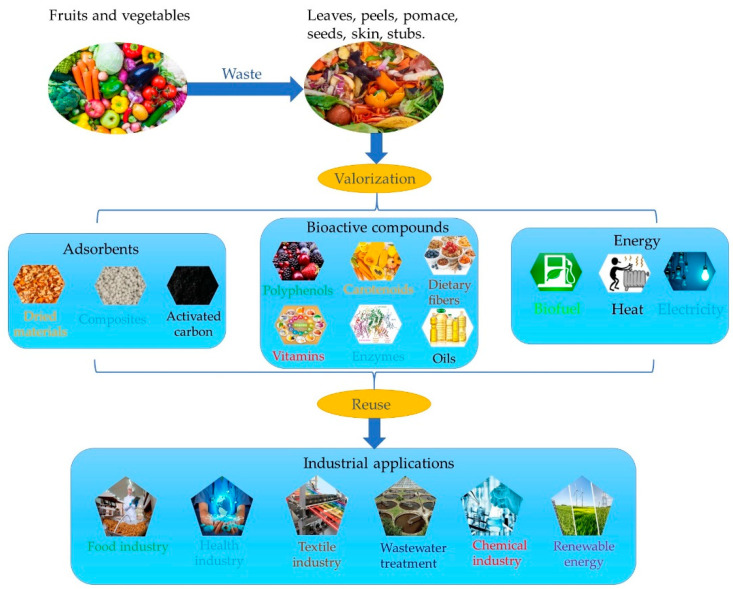
A way to minimize agri-food wastes by their conversion into valuable products.

Under these circumstances, wastage must be minimized in the agri-food sector. To achieve these goals, all these agri-food wastes have already been used as a source of fuel, livestock feeds, or organic fertilizers. Today, based on the main research and development topics, eco- and green technologies are one of the most reliable and efficient domains to sustain durability and resource conservation. Halada and Yamamoto describe the eco-materials as materials that build up a higher level of environmental protection across their life cycle without reducing their performance [50]. An example is shown in Figure 2.

Wastes are mainly made of peels, seed, pomace, and skin and these could have potential benefits as bioactive substrates. Among these, carotenoids, polyphenols, dietary fibers, enzymes, vitamins, and oils are essential in the health and food applications, or textile industry. The wastes that can be converted into bioactive compounds lead to a sustainable environment.

**Figure 3:**
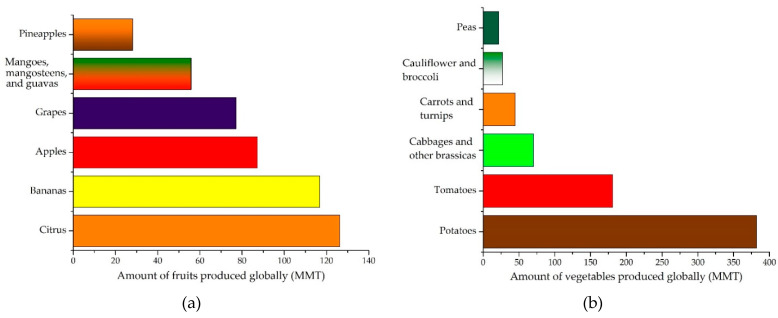
Globally produced fruits (**a**) and vegetables (**b**).

Each year, significant quantities of fruits are produced globally: 126.29 million metric tons (MMT) of citrus in the first place, 116.78 MMT of bananas as the second commodity, 87.24 MMT of apples as the third commodity, 77.14 MMT of grapes in fourth place, followed by mangoes, mangosteens, and guavas (about 55.85 MMT), and pineapples (about 28.18 MMT) [51]. Regarding vegetable production, the most important quantity is represented by potatoes (3820.00 MMT) and tomatoes (180.77 MMT), followed by cabbages and other brassicas (70.15 MMT), carrots and turnips (44.76 MMT), cauliflower and broccoli (26.91 MMT), and peas (21.77 MMT) [52]. The production of fruits and vegetables is presented in Figure 3. 

## 3. Environmental Impact of Fruit and Vegetable Wastes 

Due to the large production growth, combined with the absence of appropriate methods and infrastructure for manipulation, the waste and losses of these food commodities increased alongside their residue quantities. The FAO has assumed that at least one-third of the world’s food (estimated as 931 MMT in 2019) is lost and wasted annually (UNEP Food Waste Index Report 2021), while the horticultural commodities waste ranks first among all types of foods, reaching up to 60% [53]. These wastes result from the combined production and manipulation stages including the supply chain, classification, and ranking, marketing, storage, and processing.

Through his research, Sagar et al. highlighted that about 55 MMT of fruit and vegetable wastes are generated in developed areas from China, India, the Philippines, and the United States. These wastes result from processing, packaging, distribution, and consumption activities [49]. It was observed that an important quantity of fruit and vegetables are becoming wastes as a postharvest process before reaching the consumer and another high quantity is generated after consuming, especially in developing countries. A major threat appears concerning the decomposing of these fruit and vegetable wastes when harmful greenhouse gases are evacuated [49]. During industrial processing, fruit and vegetable wastes are produced in significant quantities; being necessary an adequate management and recycling stage is required due to their harmful environmental impact. These wastes have different characteristics morphologies and various unused and residual parts such as leaves, roots, peels, pulp, tubers, seeds, pomace, etc. [54,55]. Concerning the apple wastage, 89.09% resulted during the slicing and 10.91% consist of seed and pulp. Papaya wastage consists of about 8.5% as peel resulting from dicing, 6.5% as seeds, and 32% as useless pulp (due to the imperfection in cubes), and about 53% is represented as the final product. In the case of mandarins, the rate is about 16% peels and 84% of the final product. Pineapple generates about 15% of pulp, 15% of the top 14% of peels, 9% of the core, and 48% as the final product. The mango industry generates about 13.5% of seeds, a percentage of 18% represent the inoperable pulp, 11% as peels, and 58% as the final product [56,57]. In addition, fruit and vegetable juice preparation generates about 5.5 MMT of wastes (along with pomace). Each year, the wine and grape industry produces from 5 to 9 MMT of solid waste, and between 20% and 30% represent the processed materials [58]. Moreover, about 30% as a percentage of the leaves and stems result from canning and frozen industries, which generate about 6 MMT of solid waste each year [55].

The nature of fruit and vegetable wastes are different for each country, and additional aspects should be considered: exported transport, harvest size, processing steps, and especially energy and waters consumption as these place pressures on ecosystems. Besides these, circular economy approaches help to develop economically sustainable models and environmental protection. Del Borghi and coworkers stated in their research that the demands of the water-energy-food nexus sector are decisive on the quality of the ecosystem. These decisions are regulated within circular economy provisions especially with regard to food waste management and related to environmental degradation and climate change [59]. It is obvious that food wastes and by-products could be converted into high-added-value raw materials that contribute to environmental savings, energy, water, and greenhouse gas (GHGs) emissions abatement. Today, the total amount of GHGs emitted by a product throughout its life cycle is expressed as its carbon footprint (CF) in kilograms of equivalent CO_2_. The CF calculus for food products differs from site to site being connected with the year of production, local conditions, and variability of natural processes. Thus, different food products have different carbon intensities, some examples are presented in Table 1.

The Food and Agriculture Organization estimated that 8% of GHGs responsible for the planet’s heating are provided from food waste and half of all fruit and vegetables produced are wasted (3.7 trillion are apples) [47]. In these conditions, a GHG emissions assessment using CF is a complex procedure of food waste impact calculus. The presented examples are not comprehensive, but the overall aspects are to be considered with local specificity. For example, agri-food products are produced differently, with different resources, in different locations, and specific lifecycles. These products are shipped in different conditions, resulting in direct (transportation, production, etc.) and indirect (land use) emissions [79]. Thus, high significant uncertainties appear in GHG budgets and CF assessments. There are many studies, procedures, and methodologies developed for measuring the impact of food products as commodities and especially of resulting wastes with the specific pressures on environments.

## 4. Valorization of Agri-Food Wastes as Ecological Adsorbents

From all of the parts of unused commodities that result as wastes, peels were spotted as an ecological burden, but their lignocellulosic content encourages research for the preparation of rich biomass eco-materials, as renewable, low cost, and sustainable adsorbents for water and wastewaters treatment applications. In addition, fruit and vegetable wastes as peels are included in the containers from households in a very high percentage. Peels and skins from fruit and vegetables are a natural source, with eco-friendly and economical potential to be used as adsorbents in order to remove different pollutants from different types of water and reduce pollution, remaining a renewable and sustainable resource [5].

Today, there are plenty of treatment technologies being applied for diminishing water pollution and control of environmental quality [80]. Despite the economic features, such as high operational and maintenance costs, and also the generation of toxic sludge, the adsorption process is considered a better and low-cost option for water and wastewaters treatment. This is supported by its accessibility, variety of removed pollutants, easiness in operation, and design [5]. Activated carbon is a well-known material used as a common adsorbent both for gaseous and liquid pollutants for which the only restriction appears in terms of costs. In the last decades, various types of low-cost adsorbents were developed, characterized, and tested and the results were comprehensively studied and published [12,23,81,82,83,84,85,86,87].

A low-cost adsorbent means a material with high abundance in nature or resulted from the industry as waste with a large valorization capacity and minimum processing and the adsorption potential should be at least comparable with the one from the commercial activated carbon. However, even if the biomass from agri-food wastes could replace activated carbon as an eco-friendly material, after its treatment and activation, the research concerning their performances indicated insufficient removal potential for aquatic pollutants, thus, research is ongoing. The availability and abundance of agri-food wastes and their potential to replace natural resources exploitation with low costs are reasons for extending research and considering them as a viable option for water and waste-water treatment. Among these, ‘‘waste peels’’ from fruits and vegetables are attractive and promising eco-materials; most of the peels being wastes without any other application and with severe impact on the environment but with high potential to become a resource for water treatment technologies [5]. 

The main chemical components of fruit and vegetable wastes are lignin and cellulose that contain different functional groups from alcohols, aldehydes, ketones, carboxylic, phenolic, and ether classes [5]. Due to their polarity, these functional groups expose the capacity to link aquatic pollutants such as metals, dyes, organic compounds, usually by adsorption mechanism. Usually, the literature indicates the mass transfer process as the main step to remove pollutants by using agri-food wastes (as adsorbents), enhancing the accumulation capacity of adsorbent expressed as the pollutant quantity adsorbed, and residual pollutant concentration from solution [5,80,88]. An important aspect of adsorption efficiency is the mechanism of pollutant removal that depends on the physical and chemical characteristics of the adsorbent. In order to establish the proper mechanism, parameters as the potential rate-controlling step, kinetic models, and isotherm adsorption are studied [89,90]. The –COOH functional groups are responsible for complexation sites, whereas both –OH and –COOH groups are involved in cation exchange sites. A significant role in the adsorption process could have the ion exchange phenomena, especially when chemical modification of material takes place in order to increase the number of active binding sites and form new functional groups for pollutant removal [5,83,88]. Ionic exchange mechanism was reported in the case of the absorption process of water pollutants for lignin.

According to their chemical structure, fruit and vegetable wastes could be used only as dried materials or after advanced chemical and physical treatments [91,92,93,94,95]. The performances of wastes are based on different shapes and sizes obtained after treatment. Through physical treatment, the precursors are initially carbonized, then are subjected to an activation process with steam or CO_2_. Chemical treatment involves impregnating precursors with an activating agent, followed by a process of heating in an inert gas atmosphere [92,96,97,98,99,100]. Activating agents could dissolve the cellulose composition of the precursor and lead to the crosslinking process [100]. Chemical activation is preferred to physical activation. It requires low temperatures, produces high yields, a large area of specific surface area, involves only one step for the development of micropores, and mineral content decrease in comparison with physical activation [91,101,102,103]. One of the challenges of the chemical activation represents the washing step where corrosive agents could be used for the impurities removal [104].

## 5. Types of Pollutants

Water quality remains one of the most concerning problems of the world. Natural disasters, extreme weather events, water supply crises, even flooding, expose water resources to real challenges regarding consumption and quality. On the other side, today’s materials have to be designed according to the next generation that includes active and nanostructured materials, with attractive and advanced properties that can convert waste materials into valuable designed materials with application in water and wastewater treatment. Today, the classical treatment methods (from primary to tertiary treatments) expose the risk of producing secondary pollutants. A challenge is represented by the use of different materials with the same high efficiency and with a low concentration level of secondary pollutants generation. In this case, the use of the adsorbents from biomass represents a blueprint for the water and wastewaters—an efficient and cost-effectively treatment. The literature presents elaborated reviews with a focus on the main agricultural and non-agricultural products acting as adsorbents due to their possible mechanisms for pollutant removal. The main subjects approached by the literature are preparation methods, with the possibility of modifying waste properties for enhanced performance, mechanisms of the pollutant’s removal, re-usability options, and cost-benefit ratio, as possible future developments.

According to Younas et al., there is a real challenge regarding the treatment methods of wastewaters in correlation with the pollutant sources [41]. The authors identified two main sources, natural and derived from anthropogenic activities, emphasizing the generation sources for those pollutants that seriously affect the environment and health. Thus, the agriculture sector and households generate wastewaters containing inorganic pollutants such as heavy metals and nutrients, along with hydrocarbons, endocrine disruptors, and other organic pollutants. In addition, bacteria, viruses, and protozoa from waters are frequently identified and monitored as microbial contaminants. Together with nutrients such as phosphorus and nitrogen, water could be affected by eutrophication, which is responsible for the growth of toxin-producing cyanobacteria. 

Moreover, the effect of the presence in waters of non-biodegradable and other pollutants is their persistence in the environment for long periods of time. This leads to an accumulation in progressive levels in the biological food sequence. As a solution for these negative effects, various processes are used in order to achieve wastewater treatment before being discharged into the river body. Heavy metals and metalloids, such as Cu, Cd, Zn, Fe, Pb, HgSn, As, Al, Ag, Mn, Cr, Co, and Ni were identified in many water bodies. These elements expose higher mobility and solubility and are also persistent in the environment, leading to negative environmental and health effects. Besides these, organic pollutants (biocides, phenols, dyes, petroleum, oils, fats, proteins, starches, and medicines) affected the water quality with a significant environmental impact on ecosystem quality. Linked with these issues, many studies were carried out on the impact of heavy metals and organics on water quality, considering their harmful effects on human health. Thus, numerous advanced techniques were studied, such as adsorption on new adsorbents, membrane filtration, reverse osmosis, nanofiltration, electrodialysis, photocatalysis, phytoremediation, chemical and physical remediation, and microbial remediation processes, emphasizing the advantages and limitations in organic compounds and heavy metals removal [105,106,107,108,109,110,111,112]. Even if the efficiency of these techniques is proven by converting a large number of pollutants into harmless products, another important volume of secondary products results as wastes. In order to scale down these issues regarding waste generation, different organic and inorganic adsorbents were developed, with different adsorption capacities and efficiencies (activated carbon, clay minerals, zeolite, polymer materials, agricultural waste, etc.). The literature presents vast information about these types of materials. Focusing on agricultural wastes (as vegetables and fruits), the main parameters and efficiency data are summarized in Table 2. Due to their performances and environmental impact, even after their life-cycle, some of these materials could be associated as eco-materials, with added-value, ecofriendly, and sustainable properties.

With regard to the biggest crops, the potatoes register an increase in comparison with corn, soy, and wheat, or rice and the increase is significantly higher in the last decades. As an example, from 1968 to 2018, the increase in world potato crops was about 46.2% [125]. The peel waste from potatoes does not represent a valuable product, but it results in a large amount after the industrial processing, which can signify from 15 to 40% of the initial potato culture [126].

Potatoes are considered the second most wasted food ingredient [73]. Important research was carried out on potatoes wastes. Usually, the peel is valorized, as adsorbents after a preliminary treatment such as drying for a few days [114,115] or chemically activated with NaOH [114], ZnCl_2_ after pyrolysis [116], or H_3_PO_4_ [110,113]. All experiments indicated good efficiency removal for Cu(II), Cd(II), Ni(II), Zn(II), Mn(II), Fe(II), Co(II) heavy metals using a single element or mixture of aqueous solutions. The results were confirmed by Langmuir and Freundlich isotherm models, with the best fitting for Langmuir isotherm and the pseudo-second-order kinetic model followed [109,110]. As an overall tendency, the adsorption decreases with the increase in temperature, ionic strength, and particle size. Regarding Cu(II) recovery behavior, after five repeated cycles almost 99.5% ± 0.35 was desorbed after the last cycle [110,115].

After potatoes, tomatoes are the next significant crop. The global tomato production was estimated in 2019 at about 180.77 million tons [52]. From the processing industry, results in important tomato pomace quantities which are afterward disposed or used. The tomato pomace is made up of peels and seeds as well as fibrous matter and tomato extracts [123]. In 2019, the wastes quantities were about 14.9 million tons from 180.77 tons of tomatoes, with almost 60% wastes from seeds [58,124]. The alternative utilization of the tomato wastes could be as a functional structure for colorants, antioxidants, or other types of components with beneficial consequences on health. The tomato seeds could also represent an essential material for new products [125].

Regarding environmental application, tomato wastes were valorized as powder obtained from leaves, with good adsorption properties for Ni(II) according to the pseudo-second-order kinetic model and Langmuir isotherm [116]. Moreover, tomato waste was chemically activated with NaOH for Pb(II) adsorption with good performances of experimental data in accordance with the Freundlich isotherm and pseudo-second-order model, which shows that the process was controlled by chemisorption. Desorption studies were developed using HCl or Na_2_-EDTA [117].

From the recent FAO statistics, the global production of cabbage and other brassicas overtakes two million hectares, with almost 29 tons/ha productivity [47]. The exterior leaves represent the first by-products resulting from trimming prior to marketing the cabbage. The exterior leaves are the most exposed to contamination and are discolored due to the chemical and biochemical reactions. These are also exposed to biocides or can be affected by microbial activity, which makes them improper for consumption [126]. Cabbages leaves were studied as powder material treated at 102 °C for 24 h in order to adsorb Pb (II) and Cd (II) ions from synthetic solutions with good fitting data as Langmuir model and pseudo-second-order kinetic models suggested. Further, an excellent recovery efficiency was recorded—higher than 80% for both elements [118].

By processing, harvesting, or marketing activities, cauliflower generates between 45 and 60% (*w*/*w*) of waste. The recycling of cauliflower waste (especially represented by non-edible parts) represents a crucial issue. Great amounts of cabbage wastes are dumped directly on the ground, leading to a serious threat to the environment. Moreover, their incineration plays a significant part in the increase of CO_2_ levels [127]. Besides, bioethanol production, powder waste obtained from cauliflower was used from heavy metals removals such as Pb(II) and Cd(II) from synthetic solutions—the process is described with Langmuir and pseudo-second kinetic models [118]. By thermal treatment (pyrolysis or carbonization) the adsorption capacities of the studied material increase and ions metals such as Zn^2+^, Ni^2+^, Cd^2+^, and Cu^2+^ were efficiently removed from synthetic solutions [12]. 

In addition, good results were obtained by using slow pyrolysis at 500 °C for 6 h for cauliflower roots treatment in order to obtain a stable material for norfloxacin and chlortetracycline removal from aqueous solutions, when data were fitted with the pseudo-second-order kinetic and intra-particle diffusion model and the equilibrium experiments data were confirmed by Langmuir and Freundlich isotherm models [120]. Moreover, raw cauliflower cores were tested for Ni^2+^, Zn^2+^, Cd^2+^, and Cu^2+^ in comparison with broccoli stalks and coconut shells, the heavy metal adsorption performance being the most efficient for cauliflower [12]. 

Carrot represents one of the most significant crops with a global supply and with production of more than 37 million tons annually [2]. Their waste could represent a good source of natural compounds with potential health attributes and possible utilization in the pharmaceutical and food sectors. From the entire production, almost 30% is split after the primary processing as unused carrots or waste (CRW). CRW consist of out-graded carrots (split due to their form and properties) and processed scrap (crowns and tips resulted during separation). Some of them are partially used for animal feeding and the rest are landfilled [128].

Carrots wastes were also tested for Cr(III), Cu(II), and Zn(II) adsorption. It was observed that the adsorption took place within 10 min and the equilibrium was reached after 70 min, with the Freundlich model well-fitting the experimental data [119].

Eco-materials from fruit waste in terms of parameters and efficiency data for some pollutants removal are presented in the table below (Table 3).

Citrus comprises a large variety of fruits and some wastes of these are used with high efficiency for heavy metals removal. Usually, the solid waste of citrus consists of peel, seeds, and leaves, from which peel represents the most important waste. Only peel waste represents about 50% of the wet fruit mass. Due to their valuable compounds such as flavonoids, essential oils, polyphenols, carotenoids, fiber, sugars, ascorbic acid, and other trace elements, peel waste has huge economic value [1]. 

Among these, according to the United States Department of Agriculture Foreign Agricultural Service, global orange production for 2020/21 is estimated to grow by 3.6 MMT in comparison to the previous year, and this production will rise to 49.4 MMT. Combined with this, consumption, processing, and juice production are different and depend on fresh export, weather, and the increase in harvested areas [159].

Data show that the peels were intensively used. Orange peel, as a primary waste, contains flavonoids which serve a role as chelators for metals. Unmodified orange peels have been investigated for ion metals such as Ni(II), Co(II), Zn(II), Pb(II), and Cd(II), and metalloids such as As(III) [5,129,131,132,140]. Ni(II) removing from synthetic solutions similar to electroplating wastewater by using orange peels as an adsorbent was successfully carried out in three cycles of adsorption-desorption, in which the adsorption process pursues first-order kinetics. Schiewer and coworkers observed that adsorption kinetics depend on size material with similar performance with synthetic cation exchange resins [10].

Modified orange peels under alkaline or acidic medium were compatible for Cd(II), Zn(II), Co(II), and Ni(II) retention—the results having a good correlation with the Lagergren first-order kinetics model, in comparison with unmodified orange peels. The Langmuir and Freundlich adsorption isotherm models were in accordance with the experimental data for all the metal ions. According to the desorption results, the ion exchange process is involved in the adsorption process [5,130]. 

Also, orange peel modified with mercapto-acetic acid, after treatment with NaOH solution was used for Cu(II) and Cd(II) adsorption-desorption studies, with good results after more than five cycles of re-utilization. The process was developed in accordance with chemical adsorption followed by a pseudo-second-order kinetic model [5,89]. Another modification of orange peel was made by cross-linking with methyl acrylate in order to enhance the adsorption of Cu(II) ions from synthetic aqueous solution and electroplating wastewater. The material had a good regeneration capacity corresponding to four adsorption-desorption cycles, and the best fit for the equilibrium date was obtained with the Langmuir isotherm model [88]. In addition, a modified orange peel powder with magnetic nano-powder for Cd(II) adsorption from an electroplating simulated wastewater and aqueous solutions was successfully carried out. It was highlighted that the adsorption process follows a pseudo-second-order kinetic model [5].

Orange and grapefruit peels adsorb high quantities of uranium (U) from non-salty water. Orange and grapefruit peel incline to increase their uranium adsorption selectivity with other competitive ions: Mn, Co, Ni, Cu, Zn, Cd [141].

Orange wastes could act as ion exchangers when are modified with Ca(OH)_2_ (Ca-form) and HCl (H-form), respectively. Efficiency removal is highest for Fe (100%) and Pb (80%), and under 50% for Cd(II), Cu(II), Zn(II). with specific Langmuir-type adsorption [134].

Orange peels powder was used for the removal of different dyes in order to evaluate the adsorption capacity mechanism and establish a proper kinetic model. For example, Congo red, procion orange, acid violet 17, direct yellow, toluidine blue, direct blue, direct navy blue, direct red, and rhodamine B were tested in synthetic solutions at acidic pH values. As it is observed in Table 3, good adsorption capacities were obtained and as well as different regeneration capacities for the adsorbents as a function of the pH of the aqueous solutions and the structure of the dyes. Usually, both Langmuir and Freundlich isotherms were tested with good correlation data for these studies [5,136]. 

According to the preparation methods of these wastes, one procedure consists of the chemical activation of peel. Good results were obtained when H_3_PO_4_ was used for the removal of methylene blue and rhodamine B from synthetic solutions [5].

Organic compounds, especially biocides, were analyzed at contact with orange peel. It was observed that NaCl decreased the adsorption capacity for carbofuran from an aqueous solution, and a pseudo-second-order kinetic model described the kinetic mechanism [138]. 

Xu and coworkers established that both Langmuir and Freundlich models are in accordance with the adsorption data for furadan from aqueous solution [139].

Regarding lemons, the United States Department of Agriculture (USDA) indicates that global production in 2020/21 will slowly decrease by about 8.3 million tons due to the lower production in Argentina and the United States, in comparison to the European Union where weather conditions are favorable for lemon production [159]. Lemon peels are valuable materials for cosmetics, food, or biomedical applications and in the last years, numerous studies were developed in water remediation using lemon waste as peels. These wastes were used as a powder or after thermal treatment at 400 °C and chemical activation with H_3_PO_4_ for Cd(II), Pb(II), or Co(II) removal [5,133]. Lemon peels activation under acidic or basic conditions were studied for the cutting oil from an oil-in-water emulsion [149]. Pb(II) ions were successfully removed using cold alkali lemon peels [5], while Cd(II) when lemon peels are activated to develop some protonated sites on their surface [131]. Sweet lime peels were used for Cu(II) adsorption, experimental data giving the best correlation with the pseudo-second-order kinetics [150].

Methyl orange and Congo Red were tested as contaminants presented in an aqueous solution; data indicated a pseudo-first-order kinetic model when lemon peels are used. Also, real wastewaters, with basic pH and organic dye loads were studied with high efficiency of removal [5]. 

USDA and FAO do not make a difference between grapefruit and pomelo as commodities and the global production in 2020/21 is anticipated to increase by 6.9 million tons as a consequence of the favorable weather and expanded area in China and Mexico [159]. Within these figures, a huge quantity of wastes appears as a result of consumption and processing. 

Pomelo peel wastes were tested for methylene blue, crystal violet, and malachite green adsorption from synthetic solution with high efficiency as is presented in Table 3 [144,145,146]. 

Heavy metals as Pb(II), Cd(II), Ni(II), and Cu(II) were investigated at contact with pomelo peel untreated and depectinated and chemical activated with ZnCl_2_ and protonated, with efficiency in wastewater decontamination and synthetic solutions where the Langmuir isotherm and the pseudo-second-order rate model correspond to the adsorption process [5,142]. 

Grapefruit peel and polymerization product with formaldehyde were used for U(VI) removal, as a radioactive element, and for other competitive ions together with uranium, such as: Mn, Co, Ni, Cu, Zn, and Cd. Similar studies were taken onto the orange peel. It was observed that orange and grapefruit peel lead to an increase the uranium adsorption selectivity [141,147].

Bananas are one of the most used up and low-cost fruit in the world. In 2019, global exports of bananas reached an estimated 21 million tons, meaning a 10.2% increase compared with 2018 [160]. Rana et al. indicated that banana plants have no use after harvesting the fruit, excepting bioethanol, citric acid, lactic acid, cosmetics, fibers, bio-films, paper, bio-plastic, bio-electricity, etc. Besides these banana peels are also used as bio-sorbent for nitrites removal from drinking water, and also have antifungal and antibiotic properties [161].

For example, powder banana peels are used for heavy metals removal. Pb(II), Cd(II), Zn(II), Ni(II), Cu(II), Co(II), Cr(VI), Mn(II) from aqueous solutions can be removed using untreated, carbonized, chemically activated as alkali-hydrolyzed, acid-hydrolyzed, and bleached banana peels. Usually, the Langmuir and Freundlich isotherms describe the adsorption process, with specific performances for each metal [5,132,151,156].

Different banana peels substrates were used (natural peel, methylated, or as activated carbon) were tested for pollutants removal from a palm oil mill effluent [155]. Parameters such as color, TSS (total solid substances), COD (chemical oxygen demand), tannin, and lignin were investigated. The maximum percentage of removal was registered on banana peel-activated carbon, at a pH of 2. The pseudo-second-order model was fitted indicating that the biosorption process is based on the chemisorption mechanism. 

In addition, methylene blue was studied using banana peels unmodified and modified with caustic soda in aqueous solutions, with high efficiency and using the Langmuir and Freundlich isotherms models [152].

Phenolic compounds (hydroxytyrosol and tyrosol), atrazine, and ametrine were tested in order to evaluate the adsorption capacity in neutral pH water, river, and treated water samples. Langmuir, Freundlich, and Redlich–Peterson isotherm models provided good correlations for specific pollutants and good adsorption capacity, with chemisorption interactions at desorption experiment in case of phenolic compounds, as the values indicated in Table 3 [5,82,153,154]. 

According to the STATISTA site, in 2019 the global apple production was about 87.24 MMT in comparison with 2017 when the production was about 83.14 million tons [162]. Apple processing industries are responsible for high quantities of wastes as solids and liquids. The solids waste is known as “apple pomace“, and represents a combination of seeds, pulp, and skin, resulted from the concentrated apple juice, sweets, and jam production. Apple pomace is highly biodegradable, being a real threat to the environment, thus 20% is recovered as animal feed and 80% is landfilled, composted, or incinerated. Each option represents an important source of released greenhouse gases [163].

Apple juice residue chemical activated with NaOH can be adsorbent materials for Pb(II). The process follows a pseudo-second-order model which shows that the process is controlled by chemisorption [117]. Different functionalized apple residues were tested, for example for As, Cr and P as anions species when Zr was immobilized on apple peel [157], apple residue untreated, apple phosphate residue and apple xanthate residue for Cu(II), Zn(II) and Ni(II) [13].

Grapes are crops that grow in all countries of the world and give economic benefits than the other crops to farmers. China had a production of about 13,083,000 tons in 2017, meanwhile, South Africa had 2,032,582 tons [164]. Referring to grapes, wine production is one of the most significant sectors, with the central yield region in Europe (Italy, Spain, France, Germany, and Portugal), America (USA, Argentina, and Chile), as well as Australia, South Africa, and China. Wine production is linked with significant quantities of both organic wastes known as grape pomace (skins, pulp, branches, seeds, grape stems, and grape leaves that summarized about 1.5 kg generated per liter) and wastewater (about 75%) [165]. The wastes are connected with the production area and the physical-chemical properties of the waste vary slightly [3]. Other residues are represented by emissions of greenhouse gases (CO_2_, VOC, etc.), and inorganic wastes (bentonite clay, diatomaceous earth, and perlite) [165]. Chouchouli and coworkers indicated in their study that approximately 14.5 million tons of grape by-products are yield annually in Europe [166].

As an option for reuse of some wastes, grape skins were used for Cd(II) in aqueous solutions, but thanks to their low stability at pH 5, after 2 h of contact, these types of wastes gained high attention in biomedical and cosmetics applications [131].

Mangos are intensively used as fruits all over the world. Global production (including mangos, mangosteens, and guavas) reached 55.85 MMT in 2019, with an increasing trend in comparison with 2018, when the production was about 53.41 MMT [167]. The FAO predicted that the global production of mango will reach 65 million tons by 2028 [79]. The solid waste resulting after production, consumption, and processing consists of peels, stones, stalk, trimmings, and fibrous material [168]. Mango peel waste was tested for Cu(II), Ni(II), Zn(II) removal from aqueous solutions and electroplating wastewaters. Adsorption data were following the Langmuir adsorption isotherm model [158].

## 6. Disadvantages-Research Gaps

Besides the real advantages regarding converting some fruit and vegetable wastes into valuable eco-materials with high efficiency in pollutants removal from water and wastewaters, based on experimental data, the researchers indicated some restrictions as disadvantages in the use of these types of eco-materials as adsorbents. These features will represent the next developed research in the field that will contribute to the preparation and design of more sustainable adsorbents as eco-materials for which regeneration capacities and stability will be reliable properties. Until then, one of the restrictions is the use of untreated peel waste that increased the biochemical oxygen demand (BOD) and chemical oxygen demand (COD) values of wastewater as a consequence of soluble organic compounds dissolute from peel wastes composition [156] In the case of banana, orange, grapefruit, and apple residues the soluble substances (sugars, resins, pectins, etc.) diffuse into the water as colored substances. This structural instability, combined with their relatively low ion exchange capacity, interferes with the use of untreated eco-materials [13]. Moreover, grape residues were reported as having a tendency of dissolution at acidic pH [131]. One of the challenges is the immobilization of tannin and pectin with good results [141,169]. Even if the unmodified wastes are low-cost and available, chemically activated wastes indicated better adsorption than unmodified forms, due to the higher number of binding sites, with the possibility of ion-exchange processes and the creation of new functional groups [13,18,42,142]. Under these circumstances, thermal treatments, where porous and stable structures similar to activated carbon are obtained, can provide good performances without solubilization of material [112,113,137,170,171,172,173,174,175,176]. Some research, where fruit and vegetable wastes, especially peels, have been thermally treated or chemically activated, has indicated good stability and more reliable use in aqueous solutions, the results are summarized in this review. 

The literature indicates the importance of technical and economical features for low-cost adsorbents derived from agri-food wastes, often by comparison with activated carbon, with emphasizing the benefits and limits of agri-food wastes as adsorbents regarding the cost of preparation and regeneration [23,84,85,86,177]. A very important aspect regarding the chemical and thermal modifications for agri-food wastes, especially for fruits and vegetables, refers to the final price of the new modified materials, after treatments. The high prices reduce their advantages over conventional adsorbents (unmodified wastes) and lead to environmental pressures at the end of their life cycle. The new modified materials could be converted into toxic waste. As in other cases, research into the design and implementation of a new and stable material focuses on its functionality rather than the economic aspect. Fruit and vegetable waste as eco-materials with adsorption properties expose costs related to many factors: availability, source, treatment conditions, recycle, and lifetime use. In their work, Pyrzynska and coworkers stated that an adsorbent could be an option when regeneration is possible for sustaining a continuous flow of the treated effluent in order to decrease the cost of operation and overall maintenance [42]. Thus, the cost–benefit analysis is the most important step when agri-food waste becomes the subject of valorization as eco-materials with adsorbent properties.

As a potential improvement of environmental protection, eco-materials derived from agri-food wastes contribute to the diminishing of the environmental burden from a regeneration perspective. The choice of an adsorbent is based on these aspects. Further, the potential of its regeneration induces a reduction in costs for the whole process. In order to be available for regeneration, the mechanical strength of the waste and the type of removal mechanism are very important. According to the literature, all adsorbents have an ion-exchange system especially for heavy metals and nutrients, while the most appropriate conditions are mild to strongly acidic media. 

Acid solutions are the most common wastes in all industries and could be reused as regeneration agents. In industry, the scope of regeneration is based on valuable metals or other the recovery of components combined with the economic aspect of adsorbent reuse. Most research was developed in adsorption capacities and mechanisms with low interest for regeneration studies. In the latest years, this aspect was developed in different studies whilst emphasizing the importance of regeneration and final use disposal of spent adsorbents as potentially dangerous wastes [41]. The regeneration of the adsorbents could be a valuable benefit, their high potential of reusability leading to cost, disposal, and natural resources reductions.

Besides these issues, another challenge remains the transfer to the laboratory scale at the pilot and industry level. Most of the studies published in the literature, as they are presented in Table 2; Table 3, refer to experiments at the lab scale, using synthetic solutions. The lack of information on pilot scale or industrial level studies remains a drawback for real applications, this aspect being a common approach also for the other adsorbents or water treatment materials. 

## 7. Efficiency and Cost Comparison

Regarding the evaluation of the adsorption process as a viable option for water treatment, two basic components have to be evaluated, namely efficiency (adsorption capacity) and adsorbent cost [41]. If agri-food wastes are used as adsorbents, usually, the efficiency is related to a commercial adsorbent (activated carbon). Regarding the adsorbent costs, some aspects have to be estimated, namely, availability, source, preparation method, reuse/recycling method, transport distances, and life-cycle assessment. However, the literature indicates lower costs for the adsorbents as a result of agri-food wastage, without providing exact costs [20,178,179,180]. In the EU, is it estimated that 88 million tons of food waste are generated yearly with related costs of about 143 billion euros [26]. Future investigations have to be developed with a focus on the cost and economic benefits correlated with the eco-materials performance. As entry data, commercially activated carbon and ion exchangers prices and waste costs from some types of commodities have to be analyzed. For example, the price of activated carbon is over EUR 3.0 due to the adsorption capacity and adsorbent costs [41]. At first sight, the price for the dried citrus peels (EUR 300–600/ton) is about 100× lower than for ion exchangers’ price (EUR 30,000–50,000/ton) [131].

Moreover, it is important to emphasize that water quality changes with consumption models and new pollutants classes could appear as interferences; so the quantity and efficiency could be changed with a direct influence on the adsorbent costs. Between the lab- scale models and industrial treatment plants, the pilot-scale experiments must be undertaken in order to establish adequate operations, maintenance steps, labor costs, and also local circumstances.

A very important aspect of today’s environment is the threat from GHG emissions. The production of fruits and vegetables as agri-food commodities generates low GHG emissions, due to diesel and nitrogen fertilizers, with the lowest levels for potatoes and other roots [47].

## 8. Conclusions and Future Recommendations

Fruits and vegetables as hot-spot agri-food commodities are produced in abundance and have a huge potential for waste reuse in accordance with the circular economy concept and with a high impact on the quality of life. Their wastes are valuable for the next generation of eco-materials used in the environment, energy, biomedical application, pharmaceutics, and cosmetics industries. Good results were obtained using the fruit and vegetable wastes as major agri-food wastes for heavy metal and organic pollutants removal from aqueous solutions in laboratory-scale experiments, with the possibility in the future to apply this at the industrial level. The valorization of these wastes as eco-materials with adsorbent properties offers new perspectives for water sustainability.

The most produced fruits and vegetables all over the world are responsible for a huge quantity of waste. Usually, by landfilling these wastes a significant quantity of GHG is produced. In order to decrease the carbon footprint, their use as adsorbent materials at the nexus of water and energy represent a mandatory alternative. Moreover, these eco-materials are cost effective and easily available.

This paper summarizes, from the vast literature, the main characteristics of their application for heavy metals, organics, and micropollutants removal from waters. Only a few examples were identified with a focus on wastewaters. 

Until now, the lack of information regarding pilot-scale systems and industrial transfer represents the central disadvantage of using these cost-effective eco-materials as adsorbents for treatment technologies. Future studies will provide the proper solutions for these valuable materials. The following research is important to be developed: surface chemistry characterization of adsorbents and mechanism for a better understanding of adsorption mechanisms in wastewater treatment; pilot-scale experiments; cost benefits calculation before industrial implementation; efficiency of agri-food eco-materials as adsorbents with their integration in overall water treatment systems, including biological interactions; and end of use of these eco-materials. 

## Figures and Tables

**Table 1:** Carbon footprint during the production and generation of wastes of some fruits and vegetables.

CF of Fruits and Vegetables		CF of Generated Wastes	
Commodity	CF as CO_2_ Equiv.	Waste Data	CF as CO_2_ Equiv./kg [60]
Banana	100–200 g CO_2_ equivalents (eq) per banana [61]	30–40% peel (about 34.72–46.29 MMT, 2018) [62]	5.7
Citrus	0.07–0.64 kg CO_2_ eq/kg produced with a median value of 0.29 [63]	About 15–25,000 t waste/year [1]	2.1 as orange 0.5 as lemon 0.2 as grapefruit 0.25 other citrus
Grapes	0.846 kg CO_2_ eq/kg [64]	25% by-product/waste (as pomace, including skins, seeds from wine production) [65]	0.9
Apples	2.4 to 5 t of CO_2_ eq/ha/year (New Zealand) [66]	25% apple pomace (where: about 3% seeds, 95% skin and 1% stems) [67]	1.2
Mango	From 0.06 to 0.18 kg of CO_2_ eq/kg [68]	25% to 40% of the raw material is left as a residue (from 260.000 t) [69]	2.1 as exotic fruit
Tomatoes	Values varied between 0.1–10.1 CO_2_ eq/kg/year [70]	3–7% raw material lost as waste (where about 10% seeds ) [71].	8.2
Potatoes	0.10–0.16 kg CO_2_ eq/kg with 95% certainty for an arbitrary year and field [72]	5.8 million per day are thrown away (UK householders) [73] 15–40% peel of the initial potato mass as major waste [74]	0.3
Cabbage	0.12 kg CO_2_ eq/kg [75]	32.5% (13,406.25 t average annual) [76]	0.1
Carrots	N.D.	30% waste resulted from processing step (from 60,214 t/year in Switzerland) [77]	0.1
Cauliflower	3.67 kg/unit ha/year [78]	37.1% or 27,825 tons on-farm and 24% between the farm and the final consumer as average wastes annually [76]	0.3

**Table 2:** Examples of uses of vegetable wastes as adsorbents for water pollutants.

Vegetables	Type of Waste/Treatment Conditions	Eco-Materials Parameters	Pollutants and Adsorption Efficiency Data	References
Potatoes	Untreated potato peel, dried at 50 °C for 7 days	N.A.	Cu(II) 84.74 mg/g	[113,114]
	Chemical activated 0.5 M NaOH	N.A.	Cd(II) 90 mg/g; Cu II) 41.7 mg/g; Ni (II) 16.7 mg/g; Zn (II) 52.6 mg/g; Mn(II) 47.6 mg/g; Fe(II) 76.9 mg/g	[114,115]
	Pyrolysis and treatment with ZnCl_2_ (chemical activation) of peels	BET: 1078 m^2^/g V pores: 0.97cm^3^/g	Cu (II) 62 mg/g and 74 mg/g for 160 wt.% ZnCl_2_	[114,116]
	Thermal treatment at 400, 600, 800 °C (P400, P600), chemical activated with solution 75% (*w*/*w*) H_3_PO_4_	BET P400: 904.56 cm^2^/g, V pores: 0.726 cm^3^/g BET P600: 1041.43 cm^2^/g, V pores: 2.960 cm^3^/g	Co (II): 373 mg/g for P400 and 405 mg/g for P600	[114,117]
	Thermal activation at 600 °C (activated carbon peel (ACP))	BET 498 m^2^/g V pores 0.987 cm^3^/g	Pb (II)	[114,118]
	Potato peels as charcoal (PPC)	N.A.	Cu (II) 0.3877 mg/g (99.8%) %Recovery: 5 repeated cycles (last cycle: 99.5% ± 0.35)	[114,119]
Tomatoes	Leaf powder	BET 5.0518 m^2^/g V pores 0.003 cm^3^/g	NI (II) 58.82 mg/g	[120]
	Chemical activation with NaOH	BET: 8.83 m^2^/g V pores 0.0447 cm^3^/g	Pb (II) 152 mg/g (97%) Desorbtion studies: HCl: 53.473%, Na_2_-EDTA: 94.247%	[121]
Cabbages	Powder, 102 °C, 24 h	BET: 1.0265 m^2^/g	Pb(II) 60.57 mg/g ( 98.85%) Cd(II) 20.57 mg/g (54.32%) Recovery: Pb (II) 86.67% and Cd (II) 82.34%	[122]
Carrots		N.A.	Cr(III) 45.09 mg/g, Cu(II) 32.74, Zn(II) 29.61 mg/g.	[123]
Cauliflower	Powder 102 °C for 24 h	BET: 0.8905 m^2^/g	Pb (II): 47.63 mg/g (96.06%); Cd (II) 21.32 mg/g (81.31%). Recovery: Pb(II) 85.67% and Cd (II) 79.74%.	[122]
	Roots, slow pyrolysis, 500 °C, 6 h	BET 232.15 m^2^/g V pores 0.15 cm^3^/g	31.5 mg/g (92.3%) norfloxacin; 81.3 mg/g (93.2%) chlortetracycline	[124]
	Raw cauliflower cores (CC) as comparison with broccoli stalks (BS) and coconut shell (CS)	N.A.	Ni^2+^: 3.5 to 9.9 mg/g, Zn^2+^: 2.9 to 14.4 mg/g, Cd^2+^: 0.4 to 17.9 mg/g and Cu^2+^: 6.2 to 21.2 mg/g CS > CC > BS	[12]
	Pyrolysis or carbonization, temperature between (500 and 800 °C), during (2 and 4 h).	N.A.	Cd^2+^: 0.81–5.69 mg/g, Ni^2+^: 0.87–5.57 mg/g, Cu^2+^: 1.19–7.21 mg/g and Zn^2+^: 0.79–4.09 mg/g.	[12]
	Chemical activation, samples modifed with H_2_SO_4_ (1), H_3_PO_4_(2), NH_4_NO_3_ (3) and NH_3_ (4)	N.A.	Heavy metals: 4.47–10.13 mg/g(1); 0.80–5.67 mg/g (2); 1.41–2.58 mg/g (3); 2.05–9.86 mg/g (4).	[12]

N.A.—not available.

**Table 3:** Examples of use of vegetable wastes as adsorbents for water pollutants.

Fruits	Type of Waste/Treatment Conditions, Parameters	Pollutants and Adsorption Efficiency Data	References
Orange	Peels	Ni(II): 80 to 158 mg/g from 30 °C to 50 °C, 96% at 50 °C. Desorption with 0.05 M HCl: 95.83% (column system); 76% (batch process). Recovery studies 89% and 93.33%, respectively	[129]
Orange	Peels	As(III) 1.18 mg/g (82.45%)	[5]
Orange	Peel cellulose modified with alkali (such as NaOH, NH_4_OH, Ca(OH)_2_) and acids (such as C_6_H_6_O_7_·H_2_O, H_2_C_2_O_4_, H_3_PO_4_)	Ni(II): 1.28, Co(II): 1.23, Zn(II): 1.21 and Cd(II): 1.13 mol/kg. Desorption results with 0.05 mol/L HCl: 87.23% Zn(II), and 93.72% Cd(II). Desorption results with 0.1 mol/L HCl: 81.06% Co(II) and 80.11% Ni(II)	[5,130]
Orange	Peel modified with mercapto-acetic acid, pretreated with NaOH solution	Cu^2+^: 70.67 mg/g; Cd^2+^: 136.05 mg/g	[5,89]
Citrus	Peel	Cd(II) between 0.5 and 0.9 meq/g, according to pH values	[131]
Orange	Peel	Pb(II): 1.93 mmol/g (400 mg/g Pb)	[10]
Orange	Peel	Pb(II) 7.75 mg/g, Ni(II) 6.01 mg/g, Zn(II) 5.25 mg/g, Cu (II) 3.65 mg/g, Co(II) 1.82 mg/g; pH: 4.8–5.0.	[132]
Lemon	Peel treated at 400 °C, activated with H_3_PO_4_	Cd 96.4%, Ni 67.9%; Pb 90.11%	[133]
Orange	Wastes (as dry-gel), chemical modification with Ca(OH)_2_ (Ca-form) and washed with 0.1M HCl (H-form)	Ca-form: about 1.1 mol Pb(II), Cd(II) and Zn(II)/kg and 1.55 mol Fe(III)/kg and H-form: 2.64 mol Fe(III)/kg Efficiency removal: 100% Fe, 95% Pb, 80% Cu, 55% Cd, 40% Zn for Ca-form Efficiency removal: 98% Fe and Pb, 80% Cu, 60% Zn, 40% Cd, for H-form	[134]
Orange	Modified orange peel with methyl acrylate	Cu(II): 289.0 mg/g, pH 6.0, 94.6% Regeneration: 4 cycles (94.6% to 85.2% at the last cycle)	[88]
Orange	Saponified and modified peel with citric acid	Cd(II): 0.90 mol/kg The desorption rate: 94%, 0.15 mol/L HCl	[5,135]
Orange	Powdered peels	Congo Red: 22.4 mg/g, pH 5.0, 76.6% Procion orange: 1.3 mg/g, pH 3.0, 49% Rhodamine-B: 3.22 mg/g, pH 3.0, 67.5% Desorbtion studies: pH 12 for congo Red: 37%, pH 11 for procion orange: 78% and pH 11 for rhodamine-B: 27%.	[136]
Orange	Peel	Acid violet 17: 19.88 mg/g, pH 6.3 Maximum removal: 87% at pH 2.0 Maximum desorption: 60% at pH 10.0	[5]
		Direct Yellow DY 12 Adsorption capacity: 75.76 mg/g Efficiency removal: 96%	[5]
Orange	Peel	Navy Blue 106	[5]
Orange	Peel activated with H_3_PO_4_ BET value: 1090 m^2^/g	methylene blue and rhodamine B (114 mmol/g MB and 1.23 mmol/g RhB for Langmuir-Freundlich models)	[5,137]
Orange	Peel	Toluidine blue (TB): 314.3 mg/g. Removal: 60% at pH 3.5	[5]
Orange	Peel	Direct Red 2: 10.72 mg/g, 92%, Direct Red 80: 21.05 mg/g, 91%, pH 2. Desorption: 97.7% and 93% respectively, pH 2	[5]
Orange	Peel	Carbofuran: 84.49 mg/g at 30 °C, 44.54% for 20 mg/L	[138]
Orange	Peel	Furadan 161.29 mg/g	[139]
Orange	Peel chemical activated with KCl	Cu^2+^: 59.77 mg/g, Cd^2+^: 125.63 mg/g, Pb^2+^ 141.84 mg/g, Zn^2+^ 45.29 mg/g and Ni^2+^ 49.14 mg/g. Efficinecy, after 10 cycles: 97% (Cu^2+^), 90% (Cd^2+^) and 99% (Pb^2+^).	[5,140]
Orange	Peel powder (OPP) modified with magnetic nano-adsorbent (MNP–OPP) BET value: OPP 47.03 m^2^/g and MNP–OPP 65.19 m^2^/g.	Cd^2+^: 76.92 mg/g MNP–OPP In case of electroplating effluent: 55.38 mg/g (82%) Cd^2+^.	[5]
Orange	Peel (OP) and by polymerization with formaldehyde (OPF)	Removal U 81.2% (OP) Removal U: 96% (OPF) With other competitive ions: Mn 26.8%, Co 36.2%, Ni 41.5%, Cu 92.9%, Zn 54.9%, Cd 50.7%, U 77.9% for OP and Mn 9.1%, Co 11%, Ni 12.7%, Cu 60.5%, Zn 15.4%, Cd 15.6%, U 64.1% for OPF	[141]
Pomelo	Peels chemically acrivated (ZnCl_2_)	Pb^2+^, Cu^2+^ 90% from WWT, 21.1 mg/g from synthetic solution	[142,143]
Pomelo	Peel (PP) and depectinated pomelo peel (DPP)	Cu^2+^ adsorption capacity for PP 19.7 mg/g and DPP 21.1 mg/g at pH 4	[142]
Pomelo	Peel wastes	Methylene blue: 133mg/g (83%).	[5,144]
Pomelo	Peel wastes BET value 1357.21 m^2^/g. V tot pores 1.61 cm^3^/g	Malachite green: 178.43 mg/g. (95.06%), pH 8.0. Recovery after 4 cycles 96.35%	[5,145]
Grapefruit	peels activated ZnCl_2_	Pb^2+^ 12.73 mg/g (90% )	[5]
Grapefruit	Peels, raw and protonated	Cd^2+^: 1.7 meq/g (raw material) and 2.2 meq/g (protonated peels)	[5]
Grapefruit	Peels	crystal violet (CV): 254.16 mg/g. Efficiency 96% Recovery: 98.25% using 1 M NaOH, in repeated cycles	[5,146]
		Cd(II) Ni(II) 42.09 and 46.13 mg/g	[5]
		U (VI): 140.79 mg/g. Recovery after 3 cycles: 80%	[147]
Grapefruit	Peel (GP) and by polymerization with formaldehyde (GPF)	Removal U 77.3% (GP) Removal U: 73.4% (GPF) With other competitive ions: Mn 27.6%, Co 38.3%, Ni 43.9%, Cu 94.2%, Zn 56.6%, Cd 54.3%, U 83.3% for GP and Mn 11.5%, Co 14.2%, Ni 16.4%, Cu 65.6%, Zn 21.9%, Cd 20.3%, U 71% for GPF	[141]
Lemon	Peels	Methyl orange (MO) 50.3 mg/g, Congo red (CR) 34.5 mg/g	[148]
Lemon	Peels chemical activation (1M HCl and 1 M NaOH)	Cutting oil Adsorption capacity 8.896 mg/g, 94% at 5g/L lemon peel	[149]
Lemon	peels waste	Co: 22 mg/g	[5]
Lemon	Cold alkali peel	Pb^2+^: 630 mg/g	[5]
Lemon	Peels waste	Cd: 11.24 mg/g and efficiency removal 80.8%	[5]
Sweet lime	peels	Cu(II): 37.45 mg/g at 293 K	[150]
Lemon	Protonated peels	0.9 meq/g at pH 5	[131]
Bananas	Powder banana peels	5.71 mg/g Cd(II) and 2.18 mg Pb(II)/g Maximum removal: 89.2% for Cd(II) and 85.3% for Pb(II)	[151]
	Untreated banana peels (1), alkali-hydrolyzed banana peels (2), acid-hydrolyzed banana peels (3), and bleached Banana peels (4)	1: Cr(VI): 45% and Mn(II) 51%, 2: Cr(VI) 87% and Mn(II) 90%, 3: Cr (VI) 67% and Mn(II) 74%, 4: Cr (V) 40% and Mn(II) 67%.	[5]
	Banana peels wastes	phenolic compounds: 689 mg/g Desorbtion at neutral pH water (pH 7.3) 0.17 g/g, acetic acid (pH 1.2) 0.30 g/g and alkaline water (pH 12) 0.12 g/g phenolic compounds	[5,82]
	Banana peels (NBP) and modified with caustic soda (ABP)	methylene blue adsorption capacities: 19.671 mg/g (ABP) and 18.647 mg/g (NBP). ABP: 98.93% for pH 4–8	[152]
	Banana peel waste	atrazine 93.8% and ametryne 95.2%. Desorption of ametryne: 31.5% and 47.5% for atrazine	[153]
	Charred banana peels chemical activated with H_3_PO_4_	Atrazine: 14 mg/g. 90–99% atrazine removal	[154]
	Banana peel treated with acid, alkali, and water	Adsorption capacities: 7.97 (Pb^2+^), 6.88 (Ni^2+^), 5.80 (Zn^2+^), 4.75 (Cu^2+^), and 2.55 mg/g (Co^2+^) at pH of around 5.4–5.8	[5,132]
	Natural banana peel (1), methylated banana peel (2)	Palm oil mill effluent. (1)97 mg/g color, 25 mg/g TSS, and 90.5 mg/g COD. (2); 137.5 mg/g color, 28.5 mg/g TSS and 93 mg/g COD.	[155]
	Carbonized banana peels, chemical activation with H_2_SO_4_	Removal for Pb: 33.3%. Removal for Zn: 27.3% Removal for Cr: 77.8%	[156]
Apples	Apple juice residue chemical activated with NaOH	Pb(II) Adsorption capacity: 108 mg/g Removal: Cca 90% Desorbtion studies: HCl: 59,647%, Na_2_-EDTA: 99,809% BET values before and after activation: 7.04 and 11.13 m^2^/g. Vpores: 8.34 × 10^−3^ cc/g	[117]
	Zr immobilized apple peel	AsO_2_^−^: 15.64 mg/g, AsO_4_^3−^15.68 mg/g, Cr_2_O_7_^2−^ 25.28 mg/g, and PO_4_^3−^20.35 mg/g. Desorbtion 90% of pollutants pH 12, after 10 min	[157]
	Apple residue (AR) and Apple Phosphate residue (P), and Apple Xanthate residue (CLX)	Cu Zn Ni: 40 30 27 mg/g P-AR Cu Zn Ni: 25 15 12 mg/g CLX-AR Cu Zn NI: 10 6 5 mg/g AR	[13]
Grapes	Grape skins	Cd^2+^ metal uptake capacity 1.20 meq/g	[131]
Mango	Mango peel waste	Cu^2+^ 46.09 mg/g, Ni^2+^ 39.75 mg/g, Zn^2+^ 28.21 mg/g Removal: 89.02%, 76.40%, and 67.27% for Cu(II), Ni(II), and Zn(II) genuine electroplating effluent: Cu(II) cca 90%, Ni(II) cca 80%, Zn(II) cca 80%	[158]

## Data Availability

Data sharing not applicable.
